# Linguistic Patterns for Code Word Resilient Hate Speech Identification

**DOI:** 10.3390/s21237859

**Published:** 2021-11-25

**Authors:** Fernando H. Calderón, Namrita Balani, Jherez Taylor, Melvyn Peignon, Yen-Hao Huang, Yi-Shin Chen

**Affiliations:** 1 Institute of Information Systems and Applications, National Tsing Hua University, East District, Guang Fu Rd. Sec. 2, No. 101, Hsinchu City 300, Taiwan; fhcalderon87@gmail.com (F.H.C.); namrita.balani@moe.gov.bz (N.B.); jherez.taylor@gmail.com (J.T.); melvyn.peignon@gmail.com (M.P.); yenhao0218@gmail.com (Y.-H.H.); 2 Social Networks and Human-Centered Computing, Taiwan International Graduate Program, Institute of Information Sciences, Academia Sinica, 128, Academia Road, Sec. 2, Nankang, Taipei 115, Taiwan

**Keywords:** hate speech, social media, linguistic patterns

## Abstract

The permanent transition to online activity has brought with it a surge in hate speech discourse. This has prompted increased calls for automatic detection methods, most of which currently rely on a dictionary of hate speech words, and supervised classification. This approach often falls short when dealing with newer words and phrases produced by online extremist communities. These code words are used with the aim of evading automatic detection by systems. Code words are frequently used and have benign meanings in regular discourse, for instance, *“skypes, googles, bing, yahoos”* are all examples of words that have a hidden hate speech meaning. Such overlap presents a challenge to the traditional keyword approach of collecting data that is specific to hate speech. In this work, we first introduced a word embedding model that learns the hidden hate speech meaning of words. With this insight on code words, we developed a classifier that leverages linguistic patterns to reduce the impact of individual words. The proposed method was evaluated across three different datasets to test its generalizability. The empirical results show that the linguistic patterns approach outperforms the baselines and enables further analysis on hate speech expressions.

## 1. Introduction

The internet allows for the free flow of information, and one of its major growing pains has been the propagation of hate speech and other abusive content. Sentences like I f*cking hate **** or go back to your **** sh*thole (**Reader advisory**: We present several examples that feature hate speech and explicit content. We want to warn the reader that these examples are lifted from our dataset and are featured here for illustrative purposes only.) can be readily found even when viewing topics that should be far removed from hate speech. This creates an atmosphere that becomes uncomfortable to engage in and can have a significant impact on online discourse. It also inflicts a damaging financial and social cost on both the social network and the victims alike [1]. Additionally, the European Union has moved to enact a law that will impose hefty fines on social media networks that fail to remove flagged hate speech content within 24 h, and other offensive content within 7 days, even going as far as to hold personal staff accountable for the inaction of these companies [2]. Social networks like Twitter try to balance the need to promote free speech and the need to create a welcoming environment. The terms of service for these platforms provide guidelines on what content is prohibited. However, hate speech (HS) can be difficult to define as there are some who argue that restrictions on what constitutes HS are in fact violations of the right to free speech. The definition can also vary in terms of geographic location and the laws that can be applied. It is thus important to adhere to a rigid definition of HS in our work. We relied on the definition from the *International Covenant on Civil and Political Rights, Article 20 (2)*, which defines hate speech as *any advocacy of national, racial, or religious hatred that constitutes incitement to discrimination, hostility, or violence* [3]. In a troubling development, online communities of users that engage in HS discourse are constantly crafting new linguistic means of bypassing automatic filters. These include intentional misspellings and adapting common words to have alternative meanings, effectively softening their speech to avoid being reported and subsequently banned from the platform.

In this study, we aimed to develop a method that detects hate-speech communities while also identifying the hate speech code words that are used to avoid detection. The main challenge addressed is being able to identify such behavior without explicitly relying on hate words that do not make the best use of the context. A linguistic pattern approach that can efficiently identify hate speech instances on social media is proposed. Such a method relies on an adaptation of an unsupervised graph-based pattern extraction [4]. This pattern extraction from hate speech data can provide features that likely reflect hate speech being expressed. By using an unsupervised method, the proposed framework does not rely on annotated data or predefined dictionaries to generate the resulting patterns. The unsupervised nature of the method exploits existing word relationships and substructures learned from the training dataset.

This article presents the following contributions:

- We addressed the constant introduction of new hate speech terms with our contextual word enrichment model that learns out-of-dictionary hate speech code words.
- We identified linguistic cues used in hate speech that do not rely on the hate corpus.
- We proposed a hate speech classifier that presents superior performance across multiple datasets.

In the following sections of this manuscript, the previous literature relevant to the study is first introduced in Section 2. The methodology begins with a preliminary study on hate speech communities and code word detection presented in Section 3. With the use of code words in mind, the linguistic-pattern-based hate speech classifier in Section 4 is presented. The performance of our method is elaborated and compared in Section 5. To conclude, in Section 6, we summarize our findings.

## 2. Related Work

In the recent past, studies on social networks and anti-social behavior detection have gained interest. Anti-social behaviors can be characterized by their attacking nature and can be categorized as personal insults [5,6], cyberbullying [7,8], toxicity [9,10,11], conflict, and offensive language [7].

The ultimate goal of studying anti-social behavior is to create a safer online environment for users to freely express themselves. Previously, there has been an increase in research works related to identifying and mitigating hate speech within online social platforms [12]. The last several years have seen a surge in research related to identifying HS within online platforms, with respect to both hate speech classification and the detection of extremist communities.

A previous study [13] made use of Twitter profiles to identify and analyze the relationships between members of extremist communities, which considered cross-country interactions as well. Burnap and Williams [14] introduced the concept of othering language (the idea of differentiating groups with“us” versus “them” rhetoric) as a useful feature for HS classification. Their work lends credence to the idea that HS discourse is not limited to the presence or absence of a fixed set of words but instead relies on the context in which it appears. Waseem [15] speaks about the impact that annotators have on the underlying classification models. Another study [16] focused on identifying whether hate speech target is directed towards a specific person or entity or towards a group of people sharing a common protected characteristic. Their results show the difference in model quality when using expert versus amateur annotators.

Djuric et al. [17] adopted the *paragraph2vec*—a modification of *word2vec*—approach for classifying user comments as being either abusive or clean. This work was extended by Nobata et al. [9], which made use of features from *n*-grams and linguistic, syntactic, and distributional Semantics. These features form their model, *comment2vec*, where each comment is mapped to a unique vector in a matrix of representative words. The joint probabilities from word vectors were then used to predict the next word in a comment. The work by Mehdad and Tetreault [18] focused on character n-gram features. As our work focuses on learning the different contexts in which words appear, we utilized neural embedding approaches with *fasttext* [19] and *dependency2vec* [20].

Magu, Joshi, and Luo [21] present their work on detecting hate speech code words, which focused on the manual selection of hate speech code words. These represent words that are used by extremist communities to spread hate content without being explicit, in an effort to evade detection systems. All of the previous studies referenced here utilize either an initial bag of words (BOW) and/or annotated data, and the general consensus is that a BOW alone is not sufficient. Furthermore, if the BOW remains static, then trained models would struggle to classify less explicit HS examples; in short, we need a dynamic BOW.

To advance the work, we propose the use of hate speech community detection in order to get data that fully represent how these communities use words for hate speech. The aim of our work was to dynamically identify new *code words* that are introduced into the corpus and to minimize the reliance on a BOW and annotated data. In addition, we took this into consideration to develop a hate-speech classifier, which is resilient to such words by utilizing context-based linguistic patterns.

## 3. Code Words Identification

A preliminary study was performed to identify hate speech communities and to better understand the features of the expressions contained in them. The work leveraged existing research that confirmed the utility of using hate speech blacklists, syntactic features, and various neural embedding approaches. An overview of a community detection methodology, as well as the different types of word context, and how they can be utilized to identify possible code words is introduced. Table 1 presents the description of notations used throughout this section.

### 3.1. Extremist Community Detection

There exist words that can take on vastly different meanings depending on the way in which they are used; that is, they act as code words under different circumstances. Collecting data from extremist communities that produce hate speech content is necessary to build this representation. The search began by referring to the Extremist Files maintained by the Southern Poverty Law Center (SPLC) (https://www.splcenter.org/, accessed on 1 January 2019), a US non-profit legal advocacy organization that focuses on civil rights issues and litigation. The SPLC keeps track of prominent extremist groups and individuals within the US, including several websites that are known to produce extremist and hate content, the most prominent of these being Daily Stormer (https://www.dailystormer.com/, accessed on 1 February 2019) and American Renaissance (https://www.amren.com/, accessed on 1 January 2019). The articles on these websites are of a white supremacist nature. The two websites mentioned were selected as our seed, and we crawled their articles, storing the author name, the article body, and its title. The list of authors was then used for a manual lookup in order to tie the article author to their Twitter account. For each of these Twitter accounts extracted, their followers and friends were extracted, building a directed graph where each vertex represents a user, and edges represent a directional user–follower relationship.

**Definition** **1.** *(Vertices.) For this relationship graph g, V refers to the set containing all vertices, while $V^{\prime }$ is a random subset of V. Single-source shortest path (SSSP), which is defined as $s,t\in V^{\prime }$, the number of shortest paths from s to t, $\sigma_{st}$ was utilized. Similarly, the number of shortest paths between s and t going through v, $\sigma_{st}\left(v\right)$ is thus:*

(1) $$\forall v\in V^{\prime },g\left(v\right)=\sum_{s\neq t}\sum_{t\neq s}\frac{\sigma_{st}\left(v\right)}{\sigma_{st}}$$

The betweenness centrality [22] was computed to obtain the authors that were not directly identified from the lookup. After the initial graph processing, over three million unique users IDs were obtained. A random subset of vertices was then taken to reduce the size of the graph for computational considerations. This random subset forms a graph G containing $V_{f}$ vertices, $|V_{f}|\approx$ 20,000. Each vertex of G represents a user, while directed edges represent relationships. Consider s,t∈V. If *s* is following *t*, then a directed edge (s,t) will exist. Historical tweet data were collected from these vertices, representing over 36 million tweets. We hereafter refer to graph G as HateComm, which is our dataset that consists of the article content and titles previously mentioned in addition to the historical tweets of users within the network of author followers. *Hate speech keywords* was defined as a set of words H = $\left\{h_{1},..,h_{n}\right\}$ typically associated with hate speech in the English language. We made use of the same word source as [9]. TwitterHate refers to our dataset of tweets collected using H as seed words, while TwitterClean refers to our dataset collected without tracking any specific terms or users. The latter only collected what Twitter returned, free from the bias of collecting data based on keywords. Any tweet that contained a word w∈ H was removed.

### 3.2. Contextual Code Word Search

This study dynamically generated contextual word representations that were used for determining if a word acts as a hate speech code word or not. To create contextual word representations, the neural embedding models proposed by *dependency2vec* [20] and *fasttext* [19] were adopted. As we wished to identify out-of-dictionary words that can be linked to hate speech under a given context, as part of the pre-processing we defined a graph-based approach to reduce the word search space. Finally, the method for highlighting candidate code words is presented. The code words as well as the strength of the relationship that they may have to hate speech are reported.

#### 3.2.1. Contextual Graph Filtering

The idea for finding candidate code words was based on an approach that considers the output from the topn word list from our four embedding models, given a target word *w*. Filtering the list of possible words out of dictionary words is required to reduce the search space and obtain non-hate-speech words input to check our code word search. To achieve this, a graph construction methodology that builds a weighted directed graph of words with the output from an embedding model was devised. In this way, a graph that models word similarity or word relatedness can be constructed, depending on the embedding model we utilize.

**Figure 1:**
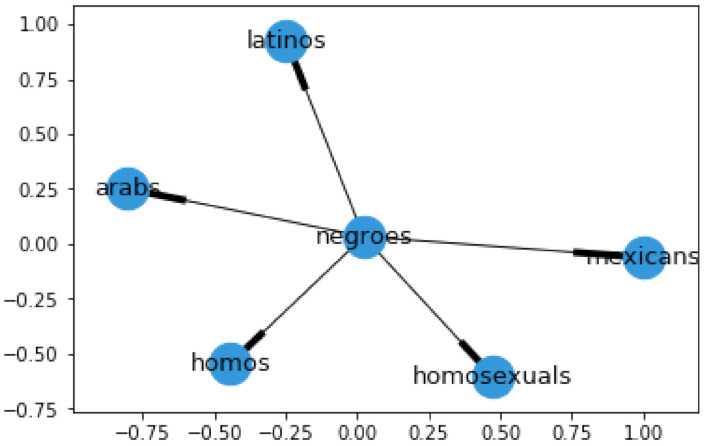
Graph ${\mathrm{CG}}_{1}$, built from word ${}_{1}$.

**Definition** **2.** *(Contextual Graph) is a weighted directed graph CG where each vertex v∈V represents a word w∈seed_input. Edges are represented by the set E. For a pair of vertices $\left(v_{1},v_{2}\right)$, an edge e∈E is created if $v_{2}$ appears in the output of simByWord, with $v_{1}$ as the input word. As an intuitive example, using $v_{1}$ = negroes from the hate speech list, the output contextual graph can be seen in Figure 1.*

To further reduce the search space, PageRank [23] was used to rank out-of-dictionary words in a graph where some of the vertices are known hate speech keywords. This allows the modeling of known hate speech words and words close to them as *important links* that pass on their weight to their successor vertices, thus boosting their importance score.

**Definition** **3.** *(boost) During the construction of any contextual graph we do a pre-initialization step where we call simByWord with a given topn for ∀w∈H if w∈$E_{vc}$. Recall that $E_{vc}$ is the stored vocabulary for the embedding model used during graph construction. The frequency of each word in the resulting collection is stored in boost. Boost(w) thus returns the frequency of the word w in this initialization step, if it exists.*

Concisely, this boosting is done to set known hate speech words as the important “pages” that pass on their weight during the PageRank computation. Edge attachment is then done via two weighting schemes that we employ.

**Definition** **4.** *(weightingScheme) Let frq(v) denote the frequency of vertex v in $E_{vc}$ for the given embedding model and $sim(v_{1},v_{2})$ the cosine similarity score for the embedding vectors under vertices $v_{1}$ and $v_{2}$. The weight wt of e($v_{1},v_{2}$) is then defined in the following:*

(2) $$wt\left(v_{1},v_{2}\right)=\left\{\begin{array}{cc} \log\left(frq\left(v_{1}\right)\right)\times boost\left(v_{1}\right)+sim\left(v_{1},v_{2}\right) & \mathrm{if}\;v_{1}\in boost\\ sim(v_{1},v_{2}) & \mathrm{if}\;v_{1}\notin boost \end{array}\right.$$

The hate speech seed graph CG then becomes a union of contextual graphs (Definition 2) created from a list of words, with a graph being created for each word. *Similarity* embedding model is used over *relatedness* for this step. The union can be seen in the following equation.

(3) $$\mathrm{CG}=\underset{w\in H}{⋃}buildGraph\left(w,D,depth,boost,topn\right)$$

PageRank was then performed on the hate speech seed graph and used the document frequency df [$df=\frac{doc\_count(w)}{N}$] for a given word *w* as a cut-off measure, where *N* is the total number of documents in a given dataset, subsequently removing all known hate speech words from the output. The assumption is that if a word *w* in our H graph is frequently used as a code word, then it should have a higher df in HateComm over TwitterClean. For the PageRank scores, we set d=0.85, as it is the standard rate of decay used for the algorithm. PR=PageRank(CG,d=0.85) and trim PR as outlined in the equation:

(4) $$\left\{\begin{array}{cc} keep(w) & \mathrm{if}\;df(w\in HateComm)>df(w\in TwitterClean)\\ remove(w) & \mathrm{if}\;df(w\in HateComm)<df(w\in TwitterClean) \end{array}\right.$$

Finally, we further refined our seed list by building a new graph using the trimmed PR+H, computing a revised PR on the resulting graph. To be clear, only the word in this list and not the actual scores were used as input for our code word search.

#### 3.2.2. Contextual Code Word Search

With our trimmed PageRank list as input, the process for selecting out-of-dictionary hate speech code words is outlined. Words were placed into categories that represent words that may be very tightly linked to known hate speech words and those that have a weaker relation.

**Definition** **5.** *(getContextRep) At the core of the method is the mixed contextual representation that we generated for an input word w from our HateComm and TwitterClean datasets. It simply gives us the word relatedness and word similarity output from embedding models trained on HateComm. The process is as follows:*

(5) $$cRep{\left(w\right)}_{HateSimilar}=simByWord\left(w,D_{H},topn\right)$$

(6) $$cRep{\left(w\right)}_{HateRelated}=simByWord\left(w,W_{H},topn\right)$$

**Definition** **6.** *(primaryCheck) accepts a word w, its contextual representation, and topn to determine if w should be placed in the primary code word bucket, returning true or false. Here, primary buckets refers to words that have some strong relation to known hate speech words. First, we calculated thresholds that check whether the number of known hate speech words in the contextual representation for a given word is above the specified threshold th.*

(7) $$th\_similarity=\left(th⩾\frac{size(HW⋂cRep_{HateSimilar})}{topn}\right)$$

(8) $$th\_relatedness=\left(th⩾\frac{size(HW⋂cRep_{HateRelated})}{topn}\right)$$

*With both thresholds, an OR operation is performed with th_check=th_similarity∨th_relatedness. Next, we determine whether w has a higher frequency in HateComm over TwitterClean by $freq\_check=\left(df(w\in HateComm)>df(w\in TwitterClean)\right)$. Finally, a word is selected as a primary code word with primary=th_check∧freq_check*

**Definition** **7.** *(secondaryCheck) accepts a word w and its contextual graph CG and searches the vertices for any v∈H, returning the predecessor vertices of v as a set if a match is found as well. We check that the set is not empty and use the truth value to indicate whether w should be placed in the secondary code word bucket. secondary=predecessor_vertices(v∈G⇒v∈H)*

### 3.3. Preliminary Findings

In order to partition our data and train our neural embeddings, first, data from Twitter were collected. Both TwitterClean and TwitterHate are composites of data collected over several time frames, including the two-week window leading up to the 2016 US Presidential Elections, the 2017 US Presidential Inauguration, and at other points during early 2017, consisting of around 10 M tweets each. In order to create HateComm, we crawled the websites obtained from the SPLC as mentioned in Section 3.1 and obtained a list of authors and attempted to link them to their Twitter profiles. This process yielded 18 unique profiles from which we collected their followers and built a graph of user:followers. We then randomly selected 20,000 vertices and collected their historical tweets, yielding around 400 K tweets. HateComm thus consists of tweets and the article contents that were collected during the scraping stage.

#### 3.3.1. Baseline Evaluation

**Figure 2:**
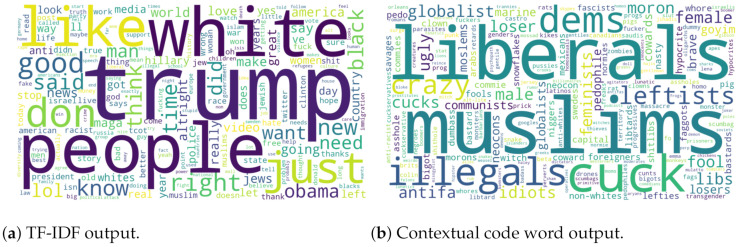
Surfaced keywords.

As a baseline benchmark, the TF-IDF word scores for HateComm were calculated and compared with frequencies of our surfaced code words. Using the TF-IDF scores is a common approach for discovering the ideas present in a corpus. For the code word weights, inverse document frequency was used. Figure 2a,b shows the difference between the TF-IDF baseline and the proposed contextual code word search. The TF-IDF output appears to be of a topical nature, particularly politics, while the code word output features multiple derogatory references throughout.

#### 3.3.2. Annotation Experiment

Throughout the work it has been stated that context is important, and an experiment was designed to reflect that. The aim was to determine if a selection of annotators would be able to identify when a given word was being used in a hate speech context without the presence of known hate speech keywords and without knowing the meaning of the code words. The experiment featured manually selected code words including one positive and one negative control word. The positive and negative samples were designed to test if annotators could identify documents that featured explicit hate speech (positive) and documents that were benign (negative).

For each dataset, an experiment was built where 10 code words (Table 2) were manually selected, and participants were asked to rate a document on a scale of very unlikely (no references to hate speech) to very likely (hate speech) (0 to 4). HateCommunity, TwitterClean, and TwitterHate were utilized as the sample pool, randomly drawing five documents for each code word (10 word × 5 documents for each experiment). Table 3 provides a sample of the documents annotators were asked to rate. Control documents were the same across all three experiments and did not feature known HS words apart from the positive control. Direct links were only provided for the experiments drawn from HateCommunity and TwitterClean. After completing these experiments, participants were given the option to move on to the TwitterHate experiment.

The experiment was designed to draw for the distinct datasets, which would reflect the use of the same word across differing situations and contexts. We obtained 52, 53, and 45 responses with Krippendorff’s alpha of K=0.871, K=0.676, and K=0.807 for HateCommunity, TwitterClean, and TwitterHate, respectively.

We evaluated if the ratings of the annotator group would reflect hate speech classification when aggregated. As we used a Likert scale for our ratings, we took ratings that were above the neutral point (2) as hate speech and ratings below as not hate speech. The precision, recall, and F1 scores can be seen in Table 4, which shows the F1 scores of 0.93 and 0.86 for HateComm and TwitterClean, respectively. These results indicate that the annotators were able to correctly classify the usage of the same word under different contexts from data that are dense in hate speech and data that reflect the general Twitter sample. This gives support to our claim that it is possible in some cases to infer hate speech intent without the presence or absence of specific words.

One of the ideas that we wanted to verify in the experiment was whether the rankings of the annotators would align with the ground truth. We include the ranking distribution for the HateComm experiment results in Table 5. The results compare the majority ranking for each word as well as the percentage against the ground truth.

## 4. Linguistic-Pattern-Based Hate Speech Identification

Considering the role of code words in hate speech, which was elaborated in the preliminary study, it provided a strong motivation to develop an automatic method for identifying hate speech that does not rely on specific terminologies. One of the shortcomings of traditional approaches in the hate speech domain is the lack of contextual information and heavily relying on annotated resources with meta-linguistic information. The advantage of using a pattern-based approach over lexicon-based approaches is the linguistic cues that can be provided to ensure resilience.

The method of extracting patterns in [4] provides a flexible representation of an underlying sentence structure. The work focuses on extracting patterns and performing multi-class emotion classification. Using such a method, we implemented an unsupervised graph approach to identify patterns that are used in hate speech. In order to fulfill the requirement of classification, the pattern extraction process was necessary. Once the patterns were extracted, the aim was to evaluate each pattern by using a ranking algorithm to assign a pattern score. This pattern score is significant as it expresses the pattern relevance to the different target categories of hate speech (HS) or not hate speech (NHS). The patterns and their scores serve as features for classification. Finally, a hate speech classifier was constructed based on a vector multiplication approach to represent tweets as a vector of the frequency of each pattern set.

### 4.1. Graph Construction

The proposed methodology requires two data sources, which are then transformed into a graph representation. These two data sources refer to opposing target classes on the classification task. For this study, we can think of them as one collection containing hate speech and another containing non-hate-speech expressions, for example, HateComm and TwitterClean, respectively.

Given the normalized datasets, each word in them is considered a token. A list of the weights of each token pair $a_{i}$ is constructed for each class: $L_{h}$ for hate speech and $L_{n}$ for non-hate-speech. Calculating the weights of each token pair is necessary, as it allows the framework to identify the underlying structures in the tweets, capturing those words that are commonly used together. For instance, a post “Build the wall higher!!” results in the following token pairs: (Build, the), (the, wall), (wall, higher), and (higher, !).

**Definition** **8.** *(Token Pair Weight) For a token pair $a_{i}\in L$, its normalized weight can be computed as shown in Equation (9).*

(9) $$w\left(a_{i}\right)=\frac{freq(a_{i})}{\max_{j\in A}freq\left(a_{j}\right)}$$

*where $freq(a_{i})$ is the frequency of token pair $a_{i}$.*

A weight aggregation is calculated to identify which of the two classes the token pairs highly represent. The goal of this step is to ensure that the weights for the token pairs represent how common they are in the specific pattern class.

**Definition** **9.** *Subsequently, new weights for arcs $a_{i}\in L_{h}$ are assigned based on a pairwise adjustment as shown in Equation (10).*

(10) $$w\left(a_{h_{i}}\right)=\left\{\begin{array}{cc} w\left(a_{h_{i}}\right)-w\left(a_{n_{j}}\right), & if\;a_{h_{j}}=a_{n_{i}}\in L_{n}\\ w\left(a_{h_{i}}\right), & otherwise \end{array}\right.$$

*A similar calculation, based on a pairwise measurement, was done for $L_{n}$, as shown in Equation (11):*

(11) $$w\left(a_{n_{i}}\right)=\left\{\begin{array}{cc} w\left(a_{n_{i}}\right)-w\left(a_{h_{j}}\right), & if\;a_{n_{j}}=a_{h_{i}}\in L_{h}\\ w\left(a_{n_{i}}\right), & otherwise \end{array}\right.$$

Arcs with high weights represent token sequences that are more common or relevant in the respective classes. Lower weights either represent tokens that are more representative of the opposite class or token sequences that are just commonly present. Furthermore, weights in $L_{h}$ and $L_{n}$ are pruned based on a threshold $\phi_{w}$.

With the extracted tokens and their weights, two weighted graphs were constructed: the hate speech graph $G_{h}\left(V_{h};A_{h}\right)$ and the non-hate-speech graph $G_{n}\left(V_{n};A_{n}\right)$. In which:

- V is a set of nodes that represents one token from the token pair.
- A is a set of vertices representing the weights for its respective token pair.

Two different graph measurements were used to determine connector words and subject words. We believe these two types of words constitute the building blocks of written expression, and they both carry out their own important functions. They are also related to the broader concepts of syntax and semantics. However, the syntactic structure can also convey meaning [24].

Connector words (CW) are those that play an important role in the syntax and structure of a text, similar to the idea of conjunction described by Halliday et al. [25]. The intuition is that these types of words are central in the graph of a corpus since they enable several connections. The eigenvector centrality was used to rank tokens and to avoid promoting very frequent words. The eigenvector centrality assigns a score to all nodes on a graph based on the idea that connections to high-scoring nodes contribute more to the score of a given node in comparison to low-scoring nodes. Nodes with an eigenvector centrality score higher than $\phi_{ec}$ were selected as connector words.

Subject words (SW) are those that can elicit a concept related to the class of the corpus. This list of words was extracted once we had obtained the list of connector words. However, taking into consideration the fact that the graph had already been pruned, we could make the assumption that the words highly connected to connector words are likely to represent information related to the topic of the graph. Opposite to connector words, subject words focus on the closeness degree of a word group. Hence, the clustering coefficient was calculated to select the words in a specified range. Nodes with a clustering score higher than $\phi_{cc}$ results were selected as subject words.

### 4.2. Pattern Extraction

The motivation of extracting linguistic patterns in comparison to a set of unigrams was to obtain features that are richer and more representative. To avoid long patterns or increase computation effort, we took into consideration patterns of two to three words. Keeping the grammatical structure of a statement intact, the patterns extracted must contain a minimum of one word from each category (CW and SW). Pattern candidate templates of two-word patterns would be extracted as follows <cw, sw> and <sw, cw>, while three-word pattern candidate templates include the following combinations: <cw, cw, sw>, <sw, cw, cw>, and <cw, sw, cw>. There are cases where a word can be marked as both CW and SW. In that case, both representations are shown. In Table 6, examples of the pattern candidate templates and what they capture are presented.

As shown, SW in the pattern examples were substituted with a wildcard “*” symbol. This operation allows flexibility of allowing other subject words, while keeping the underlying structure of the pattern intact. Additionally, this operation permits the patterns to be applied to other domains. Since our work focused on identifying linguistic cues that can be used to detect hate speech, we were interested in finding the general pattern that it represents.

### 4.3. Pattern Ranking

These linguistic patterns will act as input features into a learning model. The linguistic patterns that were extracted contain many patterns that are either too frequent in the class or not very frequent. In order to ensure we are getting patterns with substance that provide us useful information and are true representations of their respective class, a pattern ranking is crucial. To conduct this pattern ranking, a customized term frequency–inverse document frequency (TF-IDF) measure that was proposed by [4] was adopted. This method is composed of the following three measures: pattern frequency, inverse hate speech frequency, and diversity degree.

**Definition** **10.** *(Pattern frequency) The frequency of the pattern p in a collection of social data related to hate speech h. The log-scaled pattern frequency is denoted as:*

(12) pf(p,h)=log(f(p,h)+1),

*where f(p,h) is the frequency of pattern p in hate speech h.*

**Definition** **11.** *(Inverse hate speech frequency) The inverse hate speech frequency measures how common or rare the pattern p is across all hate speech collections and is computed as:*

(13) $$ihf\left(p,h\right)=\frac{\left|H\right|}{\left|\{h\in H:f(p,h)>0\}\right|}.$$

*where f(p,h) is the frequency of pattern p in hate speech h.*

**Definition** **12.** *(Diversity Degree) Diversity is based on the capturing of unique hate words in a collection by a pattern with its wildcard. If a pattern captures a wider range of subject words, their pattern diversity would rank higher. This would indicate that they are a better representation of the kind of linguistic cues used in hate speech.*

*Let div(p) denote the diversity degree of a pattern p, which is calculated as:*

(14) div(p,h)=log(dw(p,h)),

where dw(p,h) represents the number of unique words across hate speech collections that the pattern p can capture through its wildcard or placeholder “*”.

**Definition** **13.** *(Hate Degree) Finally, all three measures: pattern frequency (pf(p,h)), inverse emotion frequency (ihf(p,H)), and diversity degree (div(p)) were multiplied to form the hate degree (hd(p,h,H)).*

(15) hd(p,h,H)=pf(p,h)×ihf(p,H)×div(p,h).

However, the scope of the degree is limited by its own class. It is a true representation of the importance in its own class, but it does not take into consideration how representative it is for the other class. Thus, a degree normalization was executed:

(16) $$d_{p,h}=\left\{\begin{array}{cc} d_{p,h}\frac{d_{p,h}}{d_{p,n}} & \mathrm{if}\;p\in h\cap n\\ d_{p,h} & \mathrm{otherwise}. \end{array}\right.$$

A similar calculation was done for non-hate candidate patterns:

(17) $$d_{p,n}=\left\{\begin{array}{cc} d_{p,n}\frac{d_{p,n}}{d_{p,h}} & \mathrm{if}\;p\in n\cap h\\ d_{p,n} & \mathrm{otherwise}. \end{array}\right.$$

Patterns were pruned based on a degree threshold $\phi_{d}$. This pruning process ensures that patterns that are not representative of the class are removed. High-ranking patterns are better representations of the class in comparison to low-ranking patterns. Pattern ranking is based on the ascending rank of the degree as our work focused on generating distinct patterns that are true representations of its class. The result of this whole process is two distinct sets of ranked patterns *R* that represent each one of the classes, hate and non-hate.

### 4.4. Hate Speech Classification

Given an incoming social post, the patterns contained in it are identified to generate two frequency vectors *F* for each pattern set, $F_{h}$ for hate and *F* for non-hate.

The frequency vector for hate:

(18) $$F_{h}=\left[f_{1}^{h}f_{2}^{h}...f_{j}^{h}\right]$$

where $f_{i}$ is the frequency of pattern *i* in the post. The frequency vector for hate:

(19) $$F_{n}=\left[f_{1}^{n}f_{2}^{n}...f_{j}^{n}\right]$$

where $f_{i}$ is the frequency of pattern *i* in the post.

The classification of the post was computed:

(20) $$class=\left\{\begin{array}{cc} HateSpeech & \mathrm{if}\;R_{h}\cdot F_{h}>R_{n}\cdot F_{n}\\ Non-HateSpeech & \mathrm{otherwise}. \end{array}\right.$$

The vector whose multiplication yields the frequency vector with the higher value determines the class of the post.

## 5. Experiments and Results

To evaluate the performance of the proposed framework, a classification experiment was performed on three different datasets. If a system is to be resilient, it must demonstrate performance across different training and testing sets. Since the proposed method relies on linguistic patterns, we wanted to test its ability to identify hate speech on such patterns regardless of the specific wording contained in a training set. Additional experiments can illustrate the value in understanding the pattern usage and their resilience to code words.

### 5.1. Datasets

Detection of hate speech can be challenging, especially when trying to identify if a specific word or phrase insinuates hate speech. The collection and annotation of such data can also be difficult, as a universal definition does not exist. However, there are a few datasets that are publicly available that identify hateful, offensive, and aggressive text. The following datasets were chosen for our experiment:

- HatebaseTwitter(HbT) [26] is a Twitter dataset that consisted of 24,802 tweets. This dataset was initially built by using the Twitter API and locating tweets using a hate speech lexicon (Hatebase). This search resulted in a set of tweets, from 33,458 users, in which a timeline was extracted for each user. The timeline resulted in a set of over 85 million tweets in which a random sample of 25,000 tweets were extracted and manually coded by CrowdFlower (CF) workers. CF workers annotated the tweets into the following categories:
  1. Hate speech.
  2. Offensive but not hate speech.
  3. Neither offensive nor hate speech.
- Hatespeech-Offensive-Language(HOL). This dataset was retrieved from Kaggle, containing a set of 19,827 tweets. CF workers annotated the tweets into the following categories:
  1. Hate speech.
  2. Offensive.
  3. Neither.
- Waseem and Hovy(W&H) [10] made available a hate speech dataset from Twitter, containing a set of 16,914 tweets that were collected over the course of 2 months. They initially retrieved 136,052 tweets and annotated 16,914 of those tweets using the following categories:
  1. Racist.
  2. Sexist.
  3. Neither.

In order to validate that linguistic cues in hate speech are indeed more resilient than the hate corpus, we had to experiment with several different datasets, as mentioned above. Although these datasets vary in size and contain different characteristics of hate speech, they all belong to the Twitter platform.

### 5.2. Performance Comparison

The hate speech data limitation enforced us to adapt a 10-fold cross-validation process to ensure accurate results. The accuracy, precision, and recall were averaged after executing the cross-validation. Several baselines were implemented as detailed below.

- Traditional Baselines

In terms of traditional baselines, we considered straightforward features such as the bag of words and TF-IDF over two classification models: naive Bayes (NB) and logistic regression (LR).

- Word Embedding Baseline

Word embedding provides effective semantics for words in vector space. We used FastText [19] (FT) as our word embedding baseline as it is an efficient classification model that was proposed by research on Facebook. FastText is a strong baseline for text categorization tasks as it produces embeddings of character n-grams. Based on the embeddings, it provides predictions. The bag of words was used for classification to assist in comparison between baselines.

- State-of-the-art Baseline

Ref. [26] (SOTA) proposed a state-of-the-art feature-based classification model that incorporates distributional TF-IDF features, part-of-speech tags, and other linguistic features using support vector machines.

As observed in Table 7, the state-of-the-art model proposed by [26] shows improvements over the traditional and word embedding baselines. This can be attributed to the fact that linguistic features are considered in comparison to just word vectors or frequency counts. Our linguistic pattern approaches (LP1 LP2) in general outperformed the baselines and achieved an above-90% F1-score in two of the datasets, highlighting the performance of the proposed method. LP2 is our same pattern-based method with enriched patterns($Patterns_{e}$), as proposed by [27].

An additional advantage of relying on linguistic patterns is that we can observe the patterns our model extracted and understand which are the expressions used to convey hate speech. If an attacker were to use code words, the expression would very likely not contain any derogatory term. By relying on the context, which is obtained from the structure or syntax of the expressions, we avoid being misled by code words. Table 8 presents the top patterns across the multiple datasets to highlight how there are many expressions of hate speech that do not contain a derogatory term.

## 6. Conclusions

We proposed a dynamic method for learning out-of-dictionary hate speech code words. Our annotation experiment shows that it is possible to identify the use of words in hate speech context without knowing the meaning of the word. The results show that the task of identifying hate speech is not dependent on the presence or absence of specific keywords and supports our claim that it is an issue of context.

Considering this phenomenon, we also proposed a method to identify hate speech from social media expressions. From the understanding of code word usage, we leveraged structural patterns that do not depend on specific terms to identify hate speech. These patterns were collected in an unsupervised manner from crawled Twitter data. The experiments show that the proposed hate speech classifier can perform across different datasets; we intuit this is due to it not depending on specific terminologies.

As with many negative behaviors, hate speech is in permanent evolution. As researchers in this space, we hope to stay ahead of the trends and keep working to provide an online safe space for all users. 

## Figures and Tables

**Table 1:** Notations.

Notation	Description
CG	A contextual graph built with output from E
$D_{C}$	A dep2vec model trained on TwitterClean
$D_{H}$	A dep2vec model trained on HateComm
E	A learned embedding model of type W or D
$E_{vc}$	A stored vocabulary for a given embedding model
$W_{C}$	A word embedding model trained on TwitterClean
$W_{H}$	A word embedding model trained on HateComm

**Table 2:** Experiment selection.

Code Words	
niggers (positive control)	water (negative control)
snake	googles
cuckservatives	skypes
creatures	moslems
cockroaches	primitives

**Table 3:** Experiment Sample.

another cop killed and set on fire by googles
@user i’m sick of these worthless googles >>#backtoafrica
strange mixed-breed creatures jailed for killing white woman
germany must disinfect her land. one cockroach at a time if
necessary

**Table 4:** Aggregate annotator classification.

		Hate Speech	Not Hate Speech
	Precision	0.88	1.00
HateCommunity	Recall	1.00	0.67
	F1	0.93	0.80
	Precision	1.00	0.86
TwitterClean	Recall	0.75	1.00
	F1	0.86	0.92
	Precision	0.75	0.83
TwitterHate	Recall	0.75	0.83
	F1	0.75	0.83

**Table 5:** HateCommunity Word: Ranking Distribution.

	HateCommunity Results			
	Ground Truth		Annotators	
Words	Label	Percent	Label	Percent
niggers	Very likely	0.8	Very likely	0.68
snakes	Unlikely	0.4	Neutral	0.26
googles	Very likely	1.0	Very likely	0.41
cuckservatives	Unlikely	1.0	Likely	0.36
skypes	Likely	0.8	Likely	0.3
creatures	Very likely	0.6	Very likely	0.4
moslems	Likely	0.8	Very likely	0.39
cockroaches	Very likely	1.0	Very likely	0.40
water	Very unlikely	1.0	Very unlikely	0.65
primatives	Very likely	0.6	Very likely	0.37

**Table 6:** Examples of patterns and templates extracted through the basic pattern extraction mechanism. The asterisk (*) refers to a wildcard token which can be replaced by other subject words.

Templates	Pattern Examples
<cw,sw>	stupid *, like *, am *
<cw,cw,sw>	love you *, shut up *
<sw,cw,sw>	* for *
<sw,cw,cw>	* on the
<sw,cw>	* <hashtag>

**Table 7:** Results obtained for Twitter datasets when using different approaches. Top performance highlighted in bold.

		HbT				HOL				W&H			
Model	Features	Acc. %	Prec.%	Rec. %	F1 %	Acc. %	Prec.%	Rec. %	F1 %	Acc. %	Prec. %	Rec. %	F1 %
NB	TF-IDF	68.4	63.1	72.1	67.9	68.4	63.1	72.1	67.9	95.5	46.6	61.9	51.1
NB	BOW	86.0	39.7	58.7	42.1	86.0	51.2	77.8	54.2	61.8	37.2	72.4	57.1
LR	BOW	73.1	68.4	78.9	73.5	73.1	68.4	78.9	73.5	82.8	53.8	69.1	68.6
FT	BOW	74.0	66.7	79.1	73.3	74.0	66.7	79.1	73.3	84.7	71.7	62.0	72.8
SOTA		90.0	77.0	86.0	84.3	82.0	77.0	84.0	81.0	**90.1**	52.0	**91.0**	77.7
LP1	$Patterns_{b}$	87.9	90.0	86.8	88.2	89.0	90.5	88.5	89.3	79.8	79.0	82.7	80.5
LP2	$Patterns_{e}$	**90.4**	**92.0**	**89.6**	**90.7**	**90.8**	**92.1**	**90.3**	**91.1**	82.1	**79.9**	86.7	**82.9**

**Table 8:** Top 20 common patterns that were generated in all the datasets. “.+” represents the wildcard token.

Without Derogatory Term	With Derogatory Term
another man .+	they ass .+
.+ her man	not fucking .+
mad that .+	ass niggas .+
.+ know nothing	bitch no .+
some girls .+	faggot if .+
getting money .+	them niggas .+
makes no .+	bitch when .+
.+ has nothing	fucking with .+
funny how .+	.+ bitches be
.+ my mouth	fuck my .+
.+ come from	bitch niggas .+
.+ going down	.+ a gay
trash that .+	.+ some fucking
.+ the biggest	real nigger .+
.+ you ugly	bitch .+ URLTOK
.+ you thought	.+ faggot &
come from .+	.+ yo nigga
.+ could never	hoes .+ i
.+ stop making	hate .+ bitch
you .+ talking	.+ white bitches

## Data Availability

The different datasets used in this study were referred to throughout the manuscript and are accessible via the provided links.

## References

[1] Bloomberg (2016). Disney Dropped Twitter Pursuit Partly Over Image.

[2] Forbes (2017). Europe Fine Companies for Hate Speech.

[3] United Nations General Assembly Resolution 2200A [XX1] (1966). International Covenant on Civil and Political Rights. https://www.ohchr.org/en/professionalinterest/pages/ccpr.aspx.

[4] Argueta C., Calderon F.H., Chen Y.S. (2016). Multilingual emotion classifier using unsupervised pattern extraction from microblog data. Intell. Data Anal..

[5] Pang C. (2013). An Effective Approach for Cyberbullying Detection. Commun. Inf. Sci. Manag. Eng..

[6] Nahar V., Unankard S., Li X., Pang C. (2012). Sentiment analysis for effective detection of cyber bullying. Proceedings of the Asia-Pacific Web Conference.

[7] Burnap P., Williams M.L. (2015). Cyber Hate Speech on Twitter: An Application of Machine Classification and Statistical Modeling for Policy and Decision Making. Policy Internet.

[8] Silva L., Mondal M., Correa D., Benevenuto F., Weber I. (2016). Analyzing the Targets of Hate in Online Social Media. arXiv.

[9] Nobata C., Tetreault J., Thomas A., Mehdad Y., Chang Y. Abusive Language Detection in Online User Content. Proceedings of the 25th International Conference on World Wide Web.

[10] Waseem Z., Hovy D. (2016). Hateful Symbols or Hateful People? Predictive Features for Hate Speech Detection on Twitter. Proceedings of the NAACL Student Research Workshop.

[11] Yin D., Xue Z., Hong L., Davison B.D., Kontostathis A., Edwards L. (2009). Detection of harassment on web 2.0. Proc. Content Anal. Web.

[12] Schmidt A., Wiegand M. A Survey on Hate Speech Detection using Natural Language Processing. Proceedings of the Fifth International Workshop on Natural Language Processing for Social Media.

[13] O’Callaghan D., Greene D., Conway M., Carthy J., Cunningham P. (2013). An analysis of interactions within and between extreme right communities in social media. Ubiquitous Social Media Analysis.

[14] Burnap P., Williams M.L. (2016). Us and them: Identifying cyber hate on Twitter across multiple protected characteristics. EPJ Data Sci..

[15] Waseem Z. Are You a Racist or Am I Seeing Things? Annotator Influence on Hate Speech Detection on Twitter. Proceedings of the 2016 EMNLP Workshop on Natural Language Processing and Computational Social Science.

[16] ElSherief M., Kulkarni V., Nguyen D., Wang W.Y., Belding E. (2018). Hate Lingo: A Target-based Linguistic Analysis of Hate Speech in Social Media. arXiv.

[17] Djuric N., Zhou J., Morris R., Grbovic M., Radosavljevic V., Bhamidipati N. (2015). Hate Speech Detection with Comment Embeddings. Proceedings of the 24th International Conference on World Wide Web.

[18] Mehdad Y., Tetreault J. Do Characters Abuse More Than Words?. Proceedings of the 17th Annual Meeting of the Special Interest Group on Discourse and Dialogue.

[19] Bojanowski P., Grave E., Joulin A., Mikolov T. (2017). Enriching word vectors with subword information. Trans. Assoc. Comput. Linguist..

[20] Omer L., Yoav G. Dependency-Based Word Embeddings. Proceedings of the 52nd Annual Meeting of the Association for Computational Linguistics (Short Papers).

[21] Magu R., Joshi K., Luo J. Detecting the Hate Code on Social Media. Proceedings of the Eleventh International AAAI Conference on Web and Social Media (ICWSM 2017).

[22] Brandes U., Pich C. (2007). Centrality estimation in large networks. Int. J. Bifurc. Chaos.

[23] Page L., Brin S., Motwani R., Winograd T. (1999). The PageRank Citation Ranking: Bringing Order to the Web.

[24] Downing A., Locke P. (2006). English Grammar: A University Course.

[25] Halliday M.A.K., Matthiessen C.M., Halliday M., Matthiessen C. (2014). An Introduction to Functional Grammar.

[26] Davidson T., Warmsley D., Macy M., Weber I. Automated hate speech detection and the problem of offensive language. Proceedings of the International AAAI Conference on Web and Social Media.

[27] Saravia E., Liu H.C.T., Huang Y.H., Wu J., Chen Y.S. Carer: Contextualized affect representations for emotion recognition. Proceedings of the 2018 Conference on Empirical Methods in Natural Language Processing.

